# GSVMA: A Genetic Support Vector Machine ANOVA Method for CAD Diagnosis

**DOI:** 10.3389/fcvm.2021.760178

**Published:** 2022-02-04

**Authors:** Javad Hassannataj Joloudari, Faezeh Azizi, Mohammad Ali Nematollahi, Roohallah Alizadehsani, Edris Hassannatajjeloudari, Issa Nodehi, Amir Mosavi

**Affiliations:** ^1^Department of Computer Engineering, Faculty of Engineering, University of Birjand, Birjand, Iran; ^2^Department of Computer Sciences, Fasa University, Fasa, Iran; ^3^Institute for Intelligent Systems Research and Innovation, Deakin University, Geelong, VIC, Australia; ^4^Department of Nursing, School of Nursing and Allied Medical Sciences, Maragheh Faculty of Medical Sciences, Maragheh, Iran; ^5^Department of Computer Engineering, University of Qom, Qom, Iran; ^6^Faculty of Informatics, Technische Universität Dresden, Dresden, Germany; ^7^Faculty of Civil Engineering, TU-Dresden, Dresden, Germany; ^8^John von Neumann Faculty of Informatics, Óbuda University, Budapest, Hungary; ^9^Institute of Information Society, University of Public Service, Budapest, Hungary; ^10^Institute of Information Engineering, Automation and Mathematics, Slovak University of Technology in Bratislava, Bratislava, Slovakia

**Keywords:** coronary artery disease, genetic algorithm, support vector machine, machine learning, diagnosis

## Abstract

**Background:**

Coronary artery disease (CAD) is one of the crucial reasons for cardiovascular mortality in middle-aged people worldwide. The most typical tool is angiography for diagnosing CAD. The challenges of CAD diagnosis using angiography are costly and have side effects. One of the alternative solutions is the use of machine learning-based patterns for CAD diagnosis.

**Methods:**

Hence, this paper provides a new hybrid machine learning model called genetic support vector machine and analysis of variance (GSVMA). The analysis of variance (ANOVA) is known as the kernel function for the SVM algorithm. The proposed model is performed based on the Z-Alizadeh Sani dataset so that a genetic optimization algorithm is used to select crucial features. In addition, SVM with ANOVA, linear SVM (LSVM), and library for support vector machine (LIBSVM) with radial basis function (RBF) methods were applied to classify the dataset.

**Results:**

As a result, the GSVMA hybrid method performs better than other methods. This proposed method has the highest accuracy of 89.45% through a 10-fold crossvalidation technique with 31 selected features on the Z-Alizadeh Sani dataset.

**Conclusion:**

We demonstrated that SVM combined with genetic optimization algorithm could be lead to more accuracy. Therefore, our study confirms that the GSVMA method outperforms other methods so that it can facilitate CAD diagnosis.

## Introduction

Cardiovascular disease (CVD) is one of the most prevalent diseases which cause a lot of deaths worldwide (1). As crucial evidence for this fact, one can refer to the CVD fact sheet published by the World Health Organization (WHO), which estimated 17.9 million deaths from CVDs in 2019, representing 32% of all global deaths. Of these deaths, 85% were due to heart attack and stroke (2). An essential type of CVDs is coronary artery disease (CAD) (3). One of the reasons that made CAD such a necessary and stressful disease is the fact that nearly 25% of people who have been diagnosed with CAD died unexpectedly without any prior symptoms (4). Nowadays, electrocardiogram, cardiac stress test, coronary computed tomographic angiography, and coronary angiogram are some of the prevalent techniques used as diagnostic methods for CAD. The downside facts about all these methods are having side effects and imposing high costs on patients and health systems. Hence, today, applying machine learning methods for diagnosing CAD has become a general tendency. These techniques are important for modeling and knowledge extraction from row dataset (5).

To evaluate the performance of these new techniques, various CAD datasets have been prepared. Among these datasets, the Z-Alizadeh Sani dataset, Cleveland, and Hungarian are public.

In recent years, studies have been presented using machine learning methods for CAD diagnosis on different datasets. The well-known dataset, namely the Z-Alizadeh Sani dataset in the field of heart disease, is utilized. It is worth noting that until now, dozens of studies on the Z-Alizadeh Sani dataset have been published (5–25). The main goal of recent studies is to utilize feature selection methods to improve the accuracy of CAD diagnosis. In (10), a classification accuracy of 87.85 was obtained for CAD diagnosis by ANN classifier, so that 25 features were identified. In (14), a hybrid model titled nested ensemble nu-support vector classification method was presented to predict CAD. An accuracy of 94.66% was obtained using the hybrid method on the Z-Alizadeh Sani dataset so that 16 features were selected.

In a recent study (25), the CAD diagnosis was conducted using the weighted-average voting ensemble method. An accuracy of 98.97% was achieved using the ensemble method on five features.

We obtained the highest area under the curve (AUC) and accuracy with more valuable and important features.

The previous studies demonstrate that Support Vector Machine (SVM) performs better for binary classification and dimension reduction on a small dataset (16, 26).

Hence, we utilized the SVM method with kernel types such as analysis of variance (ANOVA), linear SVM (LSVM), and library for support vector machine (LIBSVM) with radial basis function (RBF) on the Z-Alizadeh Sani dataset. Also, a genetic algorithm as an optimizer is used to select important features in the SVM modeling process. Ultimately, among the proposed methods used in this paper, the genetic optimizer method combined with SVM and ANOVA kernel has the most accuracy of 89.45% on 31 features.

In summary, the main contributions of our paper are as follows:

1. Performing data preprocessing (transforming nominal data to numerical data and normalization)
2. Using genetic algorithm as a feature selection method for selecting important features
3. Specifying ANOVA kernel as the best kernel compared to the other kernels
4. Generating the hybrid model consist of genetic training for feature selection, SVM for classification, and a 10-fold crossvalidation technique
5. Obtaining a maximum AUC of 100% on the Z-Alizadeh Sani dataset

## Materials and Methods

**Figure 1:**
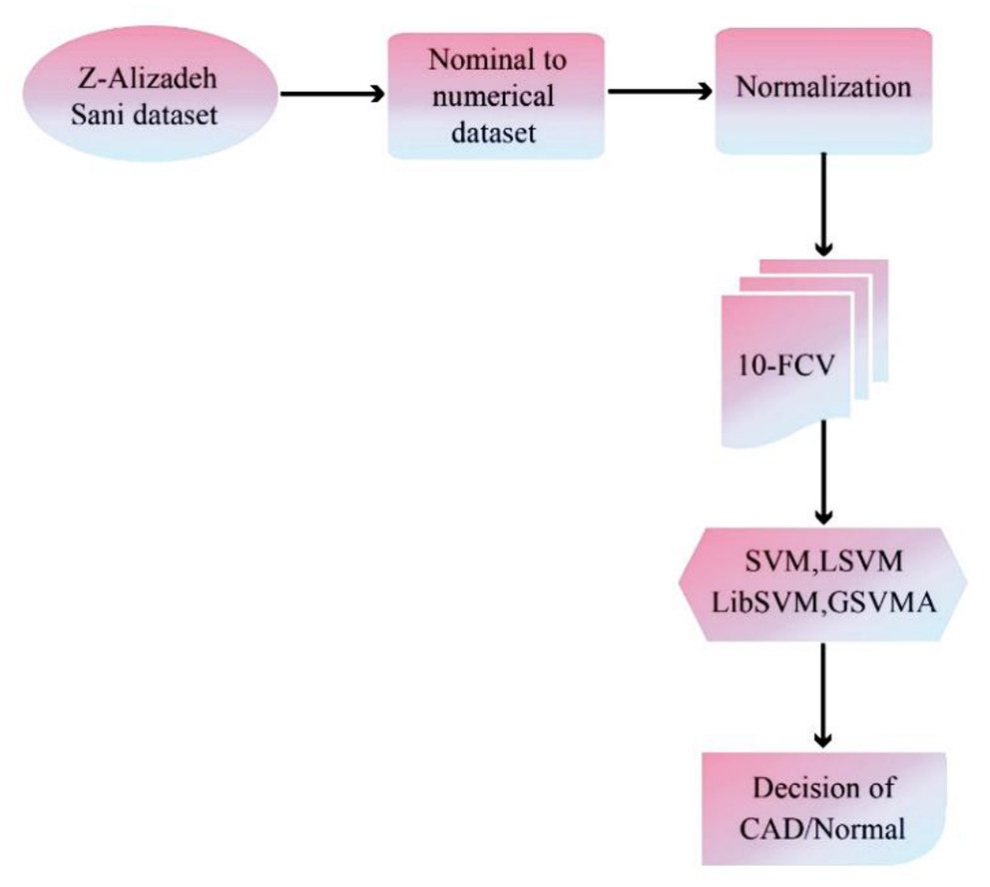
The proposed methodology framework.

The proposed methodology has been performed in three subsections. Section Z-Alizadeh Sani Dataset describes the Z-Alizadeh Sani dataset. Also, in section Data Preprocessing, data preprocessing will be done. In addition, data classification using SVM with ANOVA, LSVM, and LIBSVM with RBF and GSVMA methods is described in section Used Classification Methods. The proposed methodology framework is shown in Figure 1.

### Z-Alizadeh Sani Dataset

The Z-Alizadeh Sani dataset is one of the most common datasets used in machine learning for automatic CAD detection. This dataset is constructed from 303 patients referred to Shaheed Rajaie Cardiovascular, Medical, and Research Center^1^ A patient is categorized as a patient with CAD if one or more of his/her coronary arteries are stenosis. A coronary artery is categorized as stenosis if its diameter narrowing is ≥50% (27). Accordingly, 216 patients had CAD, and the dataset contains 88 patients with the normal situation on the Z-Alizadeh Sani. Each record in this dataset has 55 features that can be used as indicators of CAD for a patient. These features are grouped into four categories include demographic, symptom and examination, laboratory and echo, and ECG features as explained in Table 1.

**Table 1:** Description of the Z-Alizadeh-Sani dataset (5).

**Feature type**	**Feature name**	**Range**		
				**Std. error of mean**
Demographic	Age	(30–80)		0.6
Demographic	Weight	(48–120)		0.69
Demographic	Length	(140–188)		0.54
Demographic	Sex	Male, Female		—
Demographic	Body mass index (BMI) (Kb/m^2^)	(18-41)		0.24
Demographic	Diabetes mellitus (DM)	(0, 1)		0.03
Demographic	Hypertension (HTN)	(0, 1)		0.03
Demographic	Current smoker	(0, 1)		0.02
Demographic	Ex-smoker	(0, 1)		0.01
Demographic	Family history (FH)	(0, 1)		0.02
Demographic	Obesity	Yes if MBI > 25, No otherwise		—
Demographic	Chronic renal failure (CRF)	Yes, No		—
Demographic	Cerebrovascular accident (CVA)	Yes, No		—
Demographic	Airway disease	Yes, No		—
Demographic	Thyroid disease	Yes, No		—
Demographic	Congestive heart failure (CHF)	Yes, No		—
Demographic	Dyslipidemia (DLP)	Yes, No		—
Symptom and examination	Blood pressure (BP) (mmHg)	(90–190)		1.09
Symptom and examination	Pulse rate (PR) (ppm)	(50–110)		0.51
Symptom and examination	Edema	(0, 1)		0.01
Symptom and examination	Weak peripheral pulse	Yes, No		—
Symptom and examination	Lung rales	Yes, No		—
Symptom and examination	Systolic murmur	Yes, No		—
Symptom and examination	Diastolic murmur	Yes, No		—
Symptom and examination	Typical chest pain	(0, 1)		0.03
Symptom and examination	Dyspnea	Yes, No		—
Symptom and examination	Function class	1, 2, 3, 4		0.06
Symptom and examination	Atypical	Yes, No		—
Symptom and examination	Nonanginal chest pain	Yes, No		—
Symptom and examination	Exertional chest pain	Yes, No		—
Symptom and examination	Low TH Ang (low-threshold angina)	Yes, No		—
ECG	Rhythm	Sin, AF		—
ECG	Q wave	(0, 1)		0.01
ECG	ST elevation	(0, 1)		0.01
ECG	ST depression	(0, 1)		0.02
ECG	T inversion	(0, 1)		0.03
ECG	LVH (left ventricular hypertrophy)	Yes, No		—
ECG	Poor R-wave progression	Yes, No		—
Laboratory and echo	FBS (fasting blood sugar mg/dl)	(62–400)		2.99
Laboratory and echo	Cr (creatine mg/dl)	(0.5–2.2)		0.02
Laboratory and echo	TG (triglyceride mg/dl)	(37–1050)		5.63
Laboratory and echo	LDL (low-density lipoprotein mg/dl)	(18–232)		2.03
Laboratory and echo	HDL (high-density lipoprotein mg/dl)	(15–111)		0.61
Laboratory and echo	BUN (blood urea nitrogen mg/dl)	(6–52)		0.4
Laboratory and echo	ESR (erythrocyte sedimentation rate mm/h)	(1–90)		0.92
Laboratory and echo	HB (hemoglobin g/dl)	(8.9–17.6)		0.09
Laboratory and echo	K (potassium mEq/lit)	(3.0–6.6)		0.03
Laboratory and echo	Na (sodium mEq/lit)	(128–156)		0.22
Laboratory and echo	WBC (white blood cell cells/ml)	(3,700–18.000)		138.67
Laboratory and echo	Lymph (lymphocyte %)	(7–60)		0.57
Laboratory and echo	Neut (neutrophil %)	(32–89)		0.59
Laboratory and echo	PLT (platelet 1,000/ml)	(25–742)		3.49
Laboratory and echo	EF (ejection fraction %)	(15–60)		0.51
Laboratory and echo	Region with RWMA	(0–4)		0.07
Laboratory and echo	VHD (valvular heart disease)	Normal, Mild, Moderate, Severe		—
Categorical	Target classes	CAD, Normal		—

*Std: Standard*.

### Data Preprocessing

In the data analysis process, preprocessing is required after data gathering. The Z-Alizade Sani dataset was numerical and string. First, the values of features are transformed from nominal data to numerical data. The features such as sex, chronic renal failure, cerebrovascular accident, airway disease, thyroid disease, congestive heart failure, dyslipidemia, etc., are transformed. Then, the data normalization is performed. The range transformation technique is a common technique for normalizing data related to features between 0 and 1 (5). In other words, changing the range of data to zero and one means changing the mean and variance to mean zero and variance 1. Normalizing the data helps all features have an equal effect and role in diagnosing the input class so that normalizing efforts to allow all features an equal weight.

The values of features such as diabetes mellitus (DM), hypertension (HTN), current smoker, ex-smoker, etc., are transformed between zero and one. In general, normalization leads to an increase in the accuracy of the classification methods. Furthermore, a 10-fold crossvalidation (10-FCV) technique (28, 29) for partitioning the dataset was utilized so that the dataset was divided into training (90%) and test (10%) subsets. The 10-FCV process was run 10 times in which the results of the methods were obtained by averaging every 10 times.

### Used Classification Methods

#### SVM

For the first time, the SVM algorithm has been developed for data classification in (30–32), which is an optimal selection when robust predictive power is required. The SVM is a supervised machine learning algorithm that transforms data to a high dimensional space, that is, Hilbert space. Then kernel-based methods due to the visions presented by the generalization theory are exploited, and the optimization theory is performed (33). Indeed, SVM is an area parting model in which the data allocated into the support vectors are based on machine learning and model construction (34, 35).

In general, the SVM aims to find the best separator line (optimal hyperplane) between the data of the two classes so that it has the most significant possible distance from all the support vectors of the two classes. These classes are partitioned as linear and nonlinear statuses (34, 36). In these statuses, the SVM is considered that there is a set of training data *x*_1_*,x2,...,x3* €*R*^*n*^ with class *y*_1_ €*{1*,−*1}* that are binary *(x*_*i*_*, y*_*i*_*), (i*=*1, 2,...n)*, and *n* represents the number of training data points.

In this paper, we used methods such as LSVM, library SVM with RBF, SVM with ANOVA, and genetic support vector machine with ANOVA (GSVMA). RapidMiner software version 9.9 has been used to implement the methods. We described these methods in the following.

##### Linear SVM

The linear kernel is the most common kernel function for LSVM (37). The LSVM model generates an optimized hyperplane that discrete the data points of the two classes.

For an LSVM, a decision function or separator function is defined as follows:


(1) $$\begin{array}{l} f\left(x_{i}\right)=sign\left(<w,\;x_{i}>+b\right)\; \end{array}$$

= $\{\begin{array}{c} 1\;if\;y_{i}=1\\ -1\;if\;y_{i}=-1 \end{array}$

**Figure 2:**
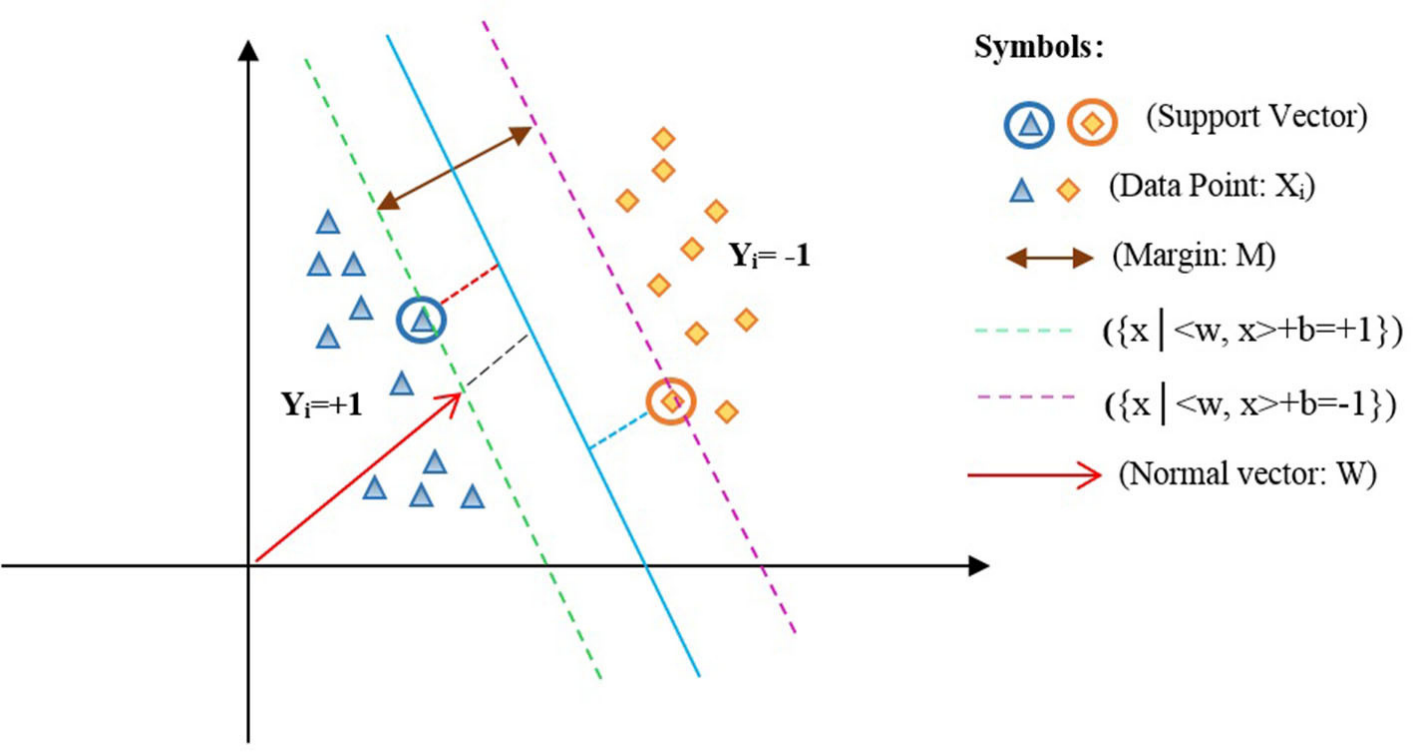
Optimized hyperplane for two-dimensional space.

In equation (1), the *w* parameter represents the weight of inertia, *b* is the width of the origin point, in which *w* €*Z* and *b* €*R*. Based on the LSVM model, an optimized hyperplane is shown in Figure 2 (34).

In Figure 2, *x*_*i*_ is the data points of the two classes labeled *y*_*i*_ =*{1*,−*1}* such that <*w,x*> +*b*=*0* represents the optimal hyperplane assigned in the average of the other two lines, that is, *{x|*<*w,x* > + *b* = +*1}* and *{x|*<*w,x* > + *b* = −*1}*. Also, *w* denotes a normal vector for the optimal hyperplane, and *b* is the offset between the hyperplane and the origin plane. Moreover, the margin *M*=*w/2* of the separator is the distance among support vectors. Therefore, the maximum margin can be obtained in the form of the following constraint optimization equation (2):


(2) $$\begin{array}{l} Minimize\;:\;\frac{1}{2}\;{\left|w\right|}^{2}subjectto\;:\;yi\left(w.x+b\right)-1\geq 0\;\forall \text{i} \end{array}$$

Based on the objective function (2), the most common approach for solving an optimization problem is to transform it into a dual problem. First, to get the dual form of the problem, the positive Lagrangian coefficients are multiplied by α_*i*_ ≥ *0* and deducted from the objective function, causing in the following equation named a primal problem (Lagrange's initial equation, *L*_*p*_):


(3) $$\begin{array}{l} {\text{L}}_{\text{p}}=\frac{1}{2}\;{\left|w\right|}^{2}\;-\;\overset{\;}{\underset{i}{\sum }}{\;\alpha \;}_{\text{i}}\;\left({\text{y}}_{\text{i}}\left(\text{w}.\text{x}+\text{b}\right)\right)-1 \end{array}$$

To solve equation (3), we are employed the Karush–Kuhn–Tucker (KKT) conditions, which perform an essential role in constraint optimization problems. These conditions state the necessary and adequate needs for the optimal solution to constraint formulas and must be a derivative of the function regarding the variables equal to zero. Exploiting KKT conditions into *L*_*p*_ has been derived from the *L*_*p*_ relation to *w* and *b*, and it sets to zero. So, the equations (4–7) are obtained as follows:


(4) $$\begin{array}{l} \frac{\partial }{\partial w_{v}}L_{p}=w_{v}-\underset{i}{\sum }\alpha_{i}y_{i}x_{iv}=0v=1,...,d \end{array}$$

(5) $$\begin{array}{l} \frac{\partial }{\partial b}L_{p}=-\underset{i}{\sum }\alpha_{i}y_{i}=0 \end{array}$$

(6) $$\begin{array}{l} \alpha_{i}\left(y_{i}\left(w.x+b\right)\right)-1\geq 0i=1,...,l\alpha_{i}\geq 0\forall_{i} \end{array}$$

(7) $$\begin{array}{l} \alpha_{i}\left(y_{i}\left(w.x+b\right)\right)-1\geq 0\forall_{i} \end{array}$$

Consequently, with the assignment of the above formulas into equation (3), equation (8) is gained.


(8) $$\begin{array}{l} Maximize\;:\;L_{D}=\underset{i}{\sum }\alpha_{i}-\frac{1}{2}\underset{i}{\sum }\underset{j}{\sum }\alpha_{i}\alpha_{j}y_{i}y_{j}\left(x_{i}.x_{j}\right) \end{array}$$

Equation (8) is named the dual problem. Hence, *L*_*p*_ and *L*_*D*_ are both obtained from the same condition. So, the optimal problem can be solved by achieving the minimum *L*_*p*_ or the maximum *L*_*p*_, the *L*_*p*_ double with the condition α_*i*_ ≥ *0*.

The parameters setting of the used LSVM method is given in Table 2.

**Table 2:** The parameters setting of the LSVM method.

**Parameters**	**Setting**
Kernel cache	200
C	0.1
Convergence epsilon	0.001
Max iterations	100,000
L pos	1.0
L neg	1.0
Balance cost	✓

The pseudocode of the LSVM is presented below.

**Algorithm 1:** The used LSVM method for CAD diagnosis.

**Input:** Extract the Z-Alizadeh Sani dataset include 303 records and 55 features.
**Output:** The diagnosed (CAD/normal) class of each test record by the generated LSVM model and determined the evaluation criteria such as accuracy, positive predictive value (PPV), F-measure, sensitivity, specificity, negative predictive value, and AUC
1: **Begin**
2: Data preprocessing: Transforming nominal records to numerical records and normalizing between zero and one
3: Divide the data using a 10-fold crossvalidation technique
4: Choose the value of the parameters setting based on Table 2
5: **While** (terminating condition is not happened by 10-fold crossvalidation) do
6: Employ LSVM train for each record
7: Generate LSVM model
8: Employ LSVM classify for testing records
9: **End while**
10: **Return** obtaining the evaluation criteria
11: **End**

##### Library SVM With RBF

The SVM is a binary classifier that it can only classify two classes. LIBSVM (38, 39) also supports the multiclass state. The difference between the two-class and multiclass problems regarding training and testing the data is that larger sets or multiclass conditions may be time-consuming. Indeed, the LIBSVM supports four different kernels by default: linear, polynomial, RBF, and sigmoid kernels, so that the RBF is the essential tool for SVM and regression classifications developed by Chavhan et al. (36). The RBF kernel is applied in the training phase (37). The advantage of using the RBF kernel is that it handles the training data to assign specified boundaries. Moreover, the RBF kernel nonlinearly maps samples into a higher-dimensional space. It can handle the training data when the relation between class labels and features is nonlinear. The RBF kernel has fewer numerical difficulties than the polynomial kernel, linear, and sigmoid.

The SVM types are selected through LIBSVM, such as the C-SVC and nu-SVC for classification tasks. Also, the epsilon-SVR and nu-SVR are used for regression tasks, and the one-class SVM is performed for distribution estimation. In this paper, the RBF kernel is selected for SVM as formulated in (9).

The most common kernel type is the RBF for SVM.


(9) $$\begin{array}{l} K\left(x_{i},x_{j}\right)=\exp\left(-{\frac{\left||x_{i}-x_{j}|\right|}{2\sigma^{2}}}^{2}\right)=\exp\left(-\gamma {\left||x_{i}-x_{j}|\right|}^{2}\right) \end{array}$$

According to (9), σ is the radial of the kernel function. Also, γ = represents the kernel parameter. The value of the kernel parameter affects the training rate and the test rate. It should be noted that the efficiency of SVM regarding the accuracy of diagnosis and generalization power is related to the situation of the penalty factor “c” and the kernel parameter “γ” (34).

Moreover, the C-SVC is used to classify data.

The parameters setting of the used LIBSVM with RBF method is described in Table 3.

**Table 3:** The parameters setting of the LIBSVM method.

**Parameters**	**Setting**
SVM type	C-SVC
Kernel type	RBF
Gamma	1.0
C	0.1
Cache size	80
Epsilon	0.001
Shrinking	✓

The pseudocode of the LIBSVM is given below.

**Algorithm 2:** The used LIBSVM method for CAD diagnosis.

**Input:** Extract the Z-Alizadeh Sani dataset include 303 records and 55 features.
**Output:** The diagnosed (CAD/normal) class of each test record and determined the evaluation criteria
1: **Begin**
2: Data preprocessing: Transforming nominal records to numerical records and normalizing between zero and one
3: Divide the data using a 10-fold crossvalidation technique
4: Choose the value of the parameters setting based on Table 3
5: **While** (terminating condition is not happened by 10-fold crossvalidation) do
6: Employ LIBSVM train for each record
7: Generate LIBSVM model
8: Employ LIBSVM classify for testing records
9: **End while**
10: **Return** obtaining the evaluation criteria
11: **End**

##### SVM With Analysis of Variance

In general, kernel types are supported by the SVM such as dot, radial, polynomial, neural, ANOVA, Epachnenikov, Gaussian combination, and multiquadric. The ANOVA kernel is defined by raised to the power “*d*” of summation of *exp(-*γ *(x-y))* where “γ” is gamma, and “*d”* is degree. The “γ” and “*d”* are regulated by the kernel gamma and kernel degree parameters, respectively. Indeed, the ANOVA kernel is also a RBF kernel. It is said to perform well in multidimensional regression and classification problems (40). The ANOVA kernel is formulated as follows:


(10) $$\begin{array}{l} k\left(x,y\right)=\overset{n}{\underset{k=1}{\sum }}\exp(-\sigma (\overset{k}{x}-\overset{k}{y}{)}^{2}{)}^{d} \end{array}$$

The parameters setting of the used SVM with the ANOVA method is given in Table 4.

**Table 4:** The parameters setting of the SVM with the ANOVA method.

**Parameters**	**Setting**
Kernel type	ANOVA
Kernel gamma	1.0
Kernel degree	2.0
Kernel cache	200
C	0.1
Convergence epsilon	0.001
Max iterations	100,000
L pos	1.0
L neg	1.0
Balance cost	✓

The pseudocode of the SVM with the ANOVA method is presented below.

**Algorithm 3:** The used SVM with ANOVA method for CAD diagnosis.

**Input:** Extract the Z-Alizadeh Sani dataset include 303 records and 55 features.
**Output:** The diagnosed (CAD/normal) class of each test record and determined the evaluation criteria
1: **Begin**
2: Data preprocessing: Transforming nominal records to numerical records and normalizing between zero and one
3: Divide the data using a 10-fold cross-validation technique
4: Choose the value of the parameters setting based on Table 4
5: **While** (terminating condition is not happened by 10-fold crossvalidation) do
6: Employ SVM with ANOVA train for each record
7: Generate SVM with ANOVA model
8: Employ SVM with ANOVA classify for testing records
9: **End while**
10: **Return** obtaining the evaluation criteria
11: **End**

##### Genetic Support Vector Machine Along With ANOVA

A genetic algorithm is a search heuristic method for solving optimization problems. This optimization algorithm uses heuristic biology techniques such as inheritance and mutation. In a genetic algorithm, to obtain the optimal response, the appropriate generation solutions are combined based on the principle of survival of the most desirable living organisms. In fact, in this algorithm, the solutions to a problem are defined in a chromosome form, consisting of a set of parameters called a gene. So, chromosomes are the proposed solutions to the problem of the genetic algorithm.

The most important application of the genetic algorithm is feature selection. Feature selection can be defined as the process of identifying related features and removing unrelated and duplicate features. The feature selection by the genetic algorithm is caused to the better efficiency of the classification methods. Hence, in this paper, we used a genetic algorithm for subset feature selection.

The stages of the genetic algorithm are as follows:

1) Initial population

The parameter of the initial population specifies the population size, that is, the number of members per generation. The genetic algorithm starts with a set of chromosomes so that several solutions with different combinations of features are randomly generated. Indeed, the chromosomes of the initial population, which are the initial solutions, comprise different combinations of the features. These combinations were randomly incorporated for each chromosome and formed the initial solutions to the problem. Hence, in this paper, using this algorithm, the best subset of the features is selected from the Z-Alizadeh Sani dataset (5). These essential features are fed to the SVM classification algorithm to classify the input dataset. In the genetic algorithm process, we set the population size to 50 and set the maximum number of generations to ten. Therefore, the size of each chromosome is related to the number of features, including 55 genes for all features.

2) Determining the fitness function

The value of each chromosome is determined by the fitness function. This function is used to examine the solutions generated in the initial population. In this paper, the fitness function is equal to accuracy, F-measure, sensitivity, specificity, PPV, and negative predictive value (NPV) (28) as determined in equations (11-16):


(11) $$\begin{array}{l} Specificity=TN/TN+FP \end{array}$$

(12) $$\begin{array}{l} Accuracy=TP+TN/TP+TN+FP+FN \end{array}$$

(13) $$\begin{array}{l} \text{Positive Predictive Value}=TP/TP+FP \end{array}$$

(14) $$\begin{array}{l} Sensitivity=TP/TP+FN \end{array}$$

(15) $$\begin{array}{l} F-measure=2*\frac{precision\;*\;Sensitivity}{precision\;+\;Sensitivity} \end{array}$$

(16) $$\begin{array}{l} \text{Negative Predictive Value}=TN/TN+FN \end{array}$$

Based on the equations (11-16), the elements of the false positive (FP), false negative (FN), true positive (TP), and true negative (TN) are described as follows:

- FP: The number of samples predicted to be positive is negative.
- FN: The number of samples predicted to be negative is positive.
- TP: The number of samples predicted to be positive is positive.
- TN: The number of samples predicted to be negative is negative.

The performance of the GSVMA method is evaluated using the mentioned fitness functions. Therefore, we tested the generated GSVMA model based on the fitness functions.

3) Selection scheme

In this stage, based on the fitness criterion, a member of a generation is selected so that members with more compatibility have more probability of making the next generation. The selection schemes such as uniform, roulette wheel (RW), stochastic universal sampling, Boltzmann, rank, tournament, and nondominated sorting exist in the genetic algorithm (6). In this paper, the RW is applied as a select scheme. Based on the scheme, the member with a higher fitness value has more probability of being selected. This scheme is one of the weighted random selection schemes. The probability of choosing each member is obtained according to the equation (17).


(17) $$\begin{array}{l} P_{i}=\frac{f_{i}}{\overset{N}{\underset{k=1}{\sum }}f_{k}} \end{array}$$

In (17), *P*_*i*_ indicates the probability of choosing the member, “*i” f*_*i*_ indicates member fitness, “*i”* and *N*, the number of members in the initial population. In this paper, the initial *P* = 0.5. The higher value of *P*_*i*_ represents that the probability of choosing the chromosome is high. In other words, this chromosome has a better chance to produce the next generation.

4) The operation of crossover

After the parent chromosomes are selected by the RW, they must be merged to generate two new children for both parents by the crossover operator. In general, there are three crossover types such as one-point, uniform, and shuffle (6, 41). Using the one-point crossover, a point on two-parent chromosomes is selected and divided into two parts so that one part of the first parent is replaced by one part of the second parent. The other type of crossover is a shuffle that two points on two-parent chromosomes are randomly selected and divided into three parts. Then, one part of the first parent is replaced by one part of the second parent, and the children in three parts are a combination of two parents. The third type of crossover is uniform. Using the uniform crossover, all the chromosome points are selected as the merge points. First, a random number between zero and one for each part of the chromosome is generated. If the generated value is less than a constant value, the genes are moved. In this paper, the shuffle has been selected as the crossover type. Also, crossover “*p”* is given 0.75.

5) Mutation action

After crossover, the mutation action is one of the essential actions to create a new generation. The mutation action is used to modify a member of the current generation to produce a new member. Due to the mutation action by random, the possibility of reaching a better member and escaping the local optimization can be efficient. In this paper, the probability value of 1.0 has been considered. This value demonstrates that mutation action is performed to create a new generation.

When the mutation action is performed, the cycle of the genetic algorithm is terminated due to convergence conditions. The convergence condition is determined based on the number of generations (number of generations = 10). Again, construction action of the new generation should be repeated (6, 41).

The parameters setting of the used genetic optimization method is given in Table 5.

**Table 5:** The parameters setting of the genetic optimization algorithm.

**Parameters**	**Setting**
Population size	50
Maximum number of generations	10
Normalize weights	✓
Plot generations	10
Draw dominated points	✓
Initial probability	0.5
Size of each chromosome = total of features	55
Probability of mutation	1.0
Crossover probability	0.75
Crossover type	Shuffle
Maximum fitness	Infinity
Fitness function	Accuracy, PPV, F-measure, sensitivity, specificity, and NPV

The pseudocode of the GSVMA method is presented below.

**Algorithm 4:** The used GSVMA method for CAD diagnosis.

**Input:** Extract the Z-Alizadeh Sani dataset include 303 records and 55 features.
**Output:** The diagnosed (CAD/normal) class of each test record and determined the 31 features as the most important features
1: **Begin**
2: Data preprocessing: Transforming nominal records to numerical records and normalizing between zero and one
3: Divide the data using a 10-fold crossvalidation technique
4: Generating a new population of members randomly (the population size= 50 and the maximum number of generations=10) based on Table 5
5: **For** i=1 to 55(chromosome size)
6: set a random of (0,1) value to gene(i) of the chromosome
7: **End For**
8: Determining the fitness function based on evaluation criteria
9: select a member of a generation (the selection scheme: uniform, roulette wheel) based on formula (16)
10: Performing crossover (crossover type: shuffle)
11: Performing mutation action (Probability of mutation=1.0)
12: **While** (terminating condition is not happened by 10-fold crossvalidation or number of generations) do
13: Execute the most important features using Genetic optimization algorithm
14: Feeding feathers SVM with ANOVA model based on algorithm 3
15: Employ SVMA classify for testing records
16: **End while**
17: **Return** obtaining the evaluation criteria and selecting the best model with 31 features
18: **End**

Based on algorithm 4, the genetic optimization method has been applied for feature selection, and the SVM with the ANOVA model has been used for classifying the dataset.

## Results

In this section, the evaluation results for the classification methods are obtained. These methods are the SVM with ANOVA, LSVM, LIBSVM with RBF, and GSVMA. Based on Table 6, accuracy (ACC), PPV, F-measure, sensitivity, specificity, NPV, and AUC had been achieved by the confusion matrix. In this paper, we have used RapidMiner Studio version 9.9 to implement the methods in the CAD diagnosis and classification process.

**Table 6:** Confusion matrix for diagnosis of CAD.

**The predicted class**	**The actual class**	
	**CAD**	**Normal**
Positive	True positive	False positive
Negative	False negative	True negative

The evaluation criteria of the methods were obtained based on equations (11-16) (28).

**Figure 3:**
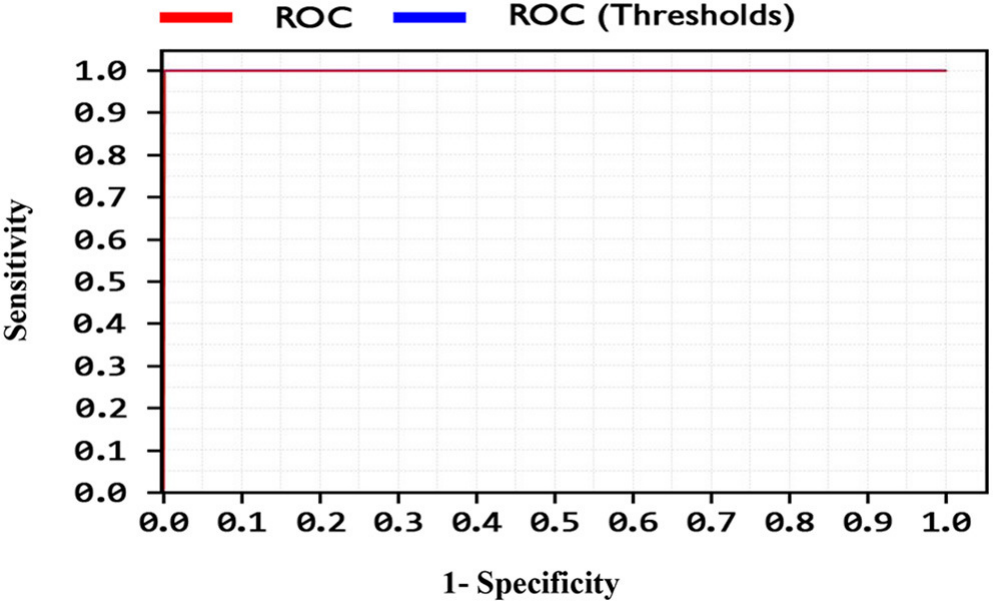
ROC curve for GSVMA method.

By comparing the performance of the methods, the ACC rates of the SVM with ANOVA, LSVM, and LIBSVM with RBF were achieved 85.13, 86.11, and 84.78%, respectively, whereas the ACC rate of the GSVMA method is 89.45% based on the 10-FCV technique. According to the other criteria, the GSVMA method had the highest PPV, F-measure, sensitivity, and specificity. Moreover, another crucial criterion used to determine the classification methods is the AUC criterion. The AUC indicates the measure of the area under the Receiver Operating Characteristic (ROC). In other words, the AUC transforms the ROC curve into a numeric demonstration of performance for classification models. The AUC of the GSVMA method is obtained 100%. The results of the evaluation criteria for methods through the 10-FCV are indicated in Table 7. Also, the ROC curve was illustrated for the GSVMA method in Figure 3.

**Table 7:** The comparison of the methods based on the Z-Alizadeh Sani dataset in this study.

**Methods**	**ACC(%)**	**PPV(%)**	**F-measure (%)**	**Sensitivity (%)**	**Specificity(%)**	**NPV(%)**	**AUC(%)**
SVM with ANOVA	85.13	80.24	72.01	69.19	91.62	68.97	89
Linear SVM	86.11	77.21	75.55	74.75	90.71	74.71	92.4
LIBSVM with RBF	84.78	76.24	72.38	70.03	90.74	70.11	82.1
**GSVMA**	**89.45**	**100**	**80.49**	**81.22**	**100**	**92.9**	**100**

*Bold the best values of the proposed method (GSVMA) to show our method is the best*.

Based on Figure 3, the ROC diagram for the GSVMA method demonstrates that the AUC value = 100%.

**Figure 4:**
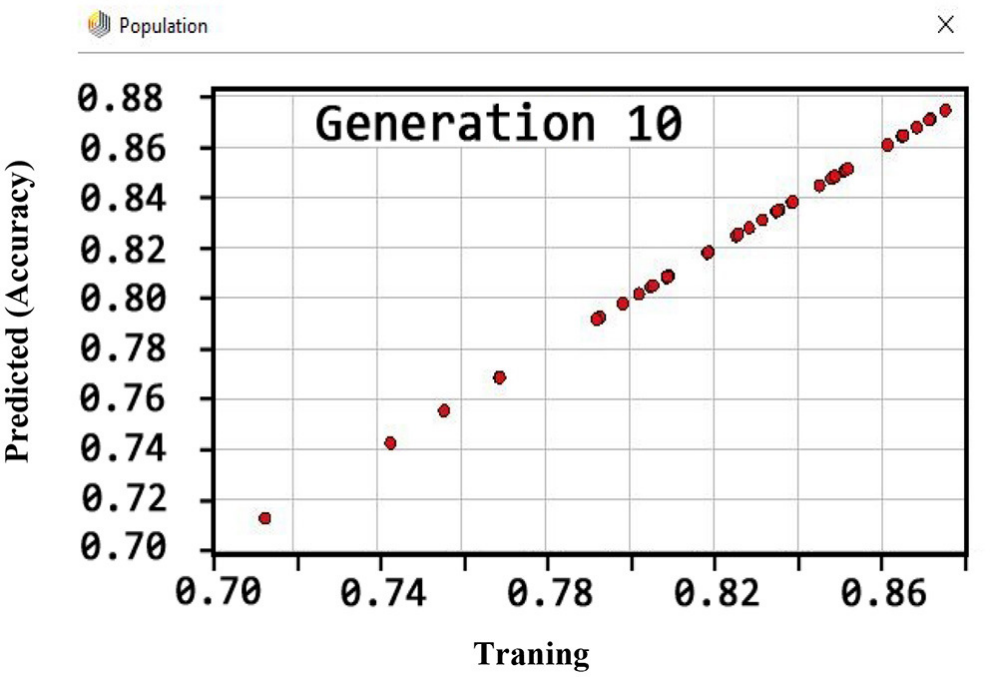
ACC diagram for GSVMA method for generation 10.

**Figure 5:**
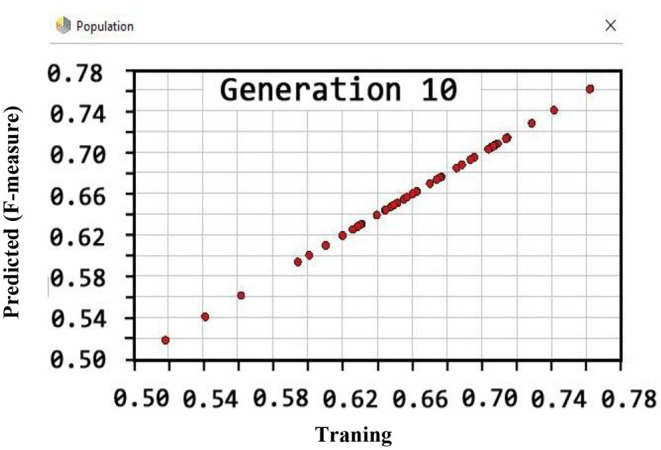
F-measure diagram for GSVMA method for generation 10.

**Figure 6:**
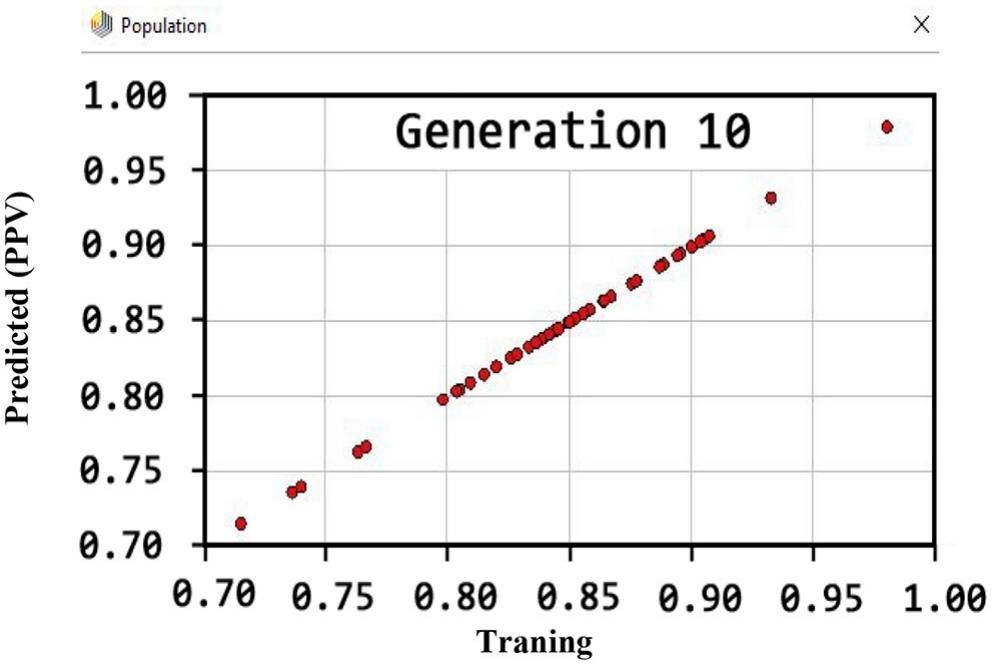
PPV diagram for GSVNA method for generation 10.

**Figure 7:**
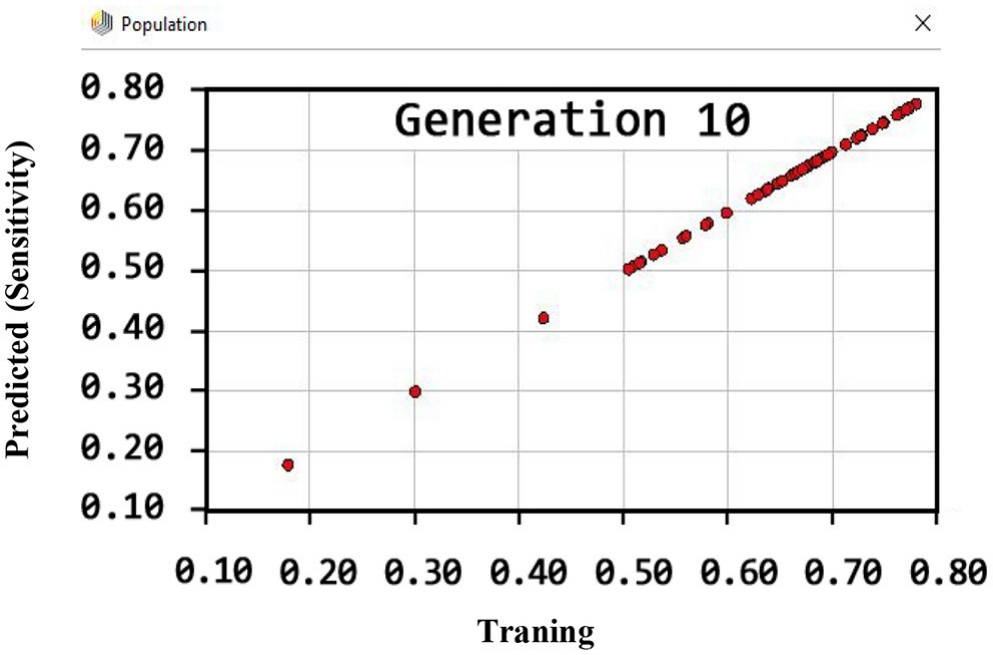
Sensitivity diagram for GSVMA method for generation 10.

**Figure 8:**
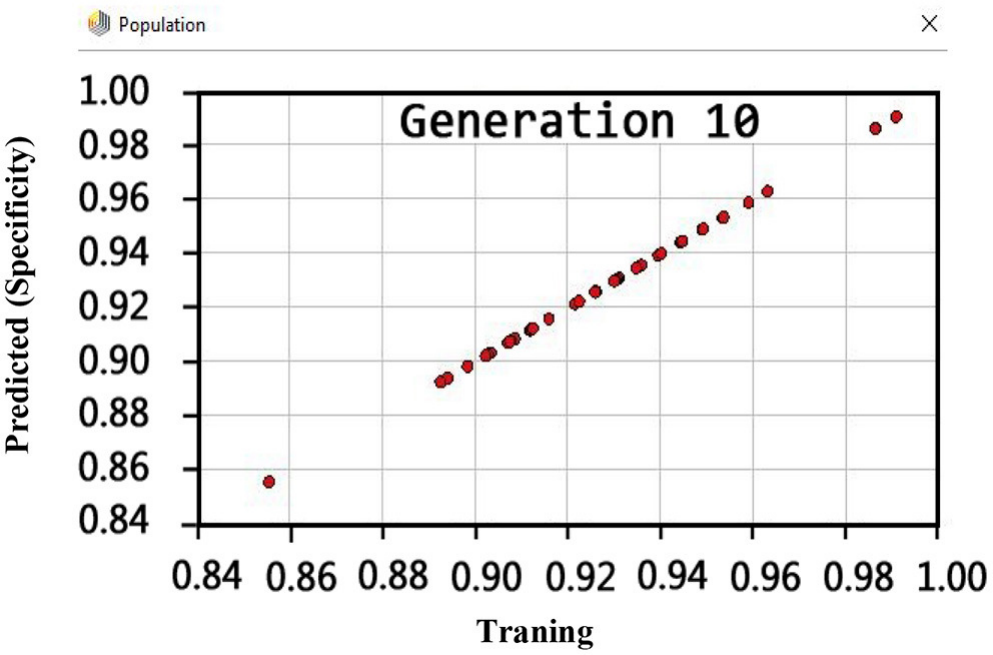
Specificity diagram for GSVNA method for generation 10.

**Figure 9:**
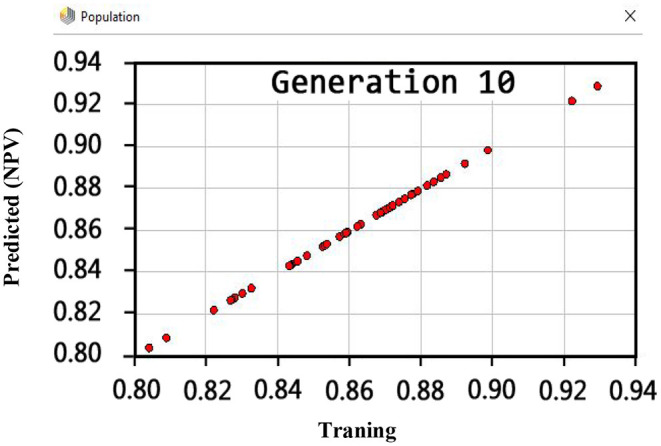
NPV diagram for GSVNA method for generation 10.

Also, the criteria of the ACC, F-measure, PPV, sensitivity, specificity, and NPV are illustrated in Figures 4–**9**, respectively. Moreover, in the 10-FCV technique, the GSVMA method was trained for 10 generations on the Z-Alizadeh Sani dataset. To find the optimal response of the evaluation criteria (fitness function = criteria), a set of initial responses are generated in each generation so that the set of responses converges toward the optimal response. In this study, convergence is related to the tenth generation. Therefore, the mentioned criteria were obtained based on the best generation (generation 10).

According to Figure 4, the ACC rate has obtained more than 88% (89.45%) through the GSVMA method for generation 10.

Based on Figure 5, the performance of the GSVMA method achieved F-measure of 80.49% for generation 10.

Figure 6 shows that the PPV has reached a maximum of 100% for the tenth generation.

By observing Figure 7, it can be inferred that the sensitivity has been more than 80% (81.22) using the GSVMA method for the tenth generation.

Regarding the specificity criterion, Figure 8 shows the maximum value of 100% using the GSVMA method for the tenth generation.

Another crucial evaluation criterion is the NPV in clinical practice. The NPV is the probability that subjects with a negative test rightly have healthy. The NPV was obtained on average 92.9% as shown in Figure 9. Similarly, three methods such as LIBSVM with RBF, LSVM, and SVM with ANOVA have been applied to the Z-Alizadeh Sani dataset. We presented the results of these methods in Table 7. Based on Table 7, the accuracy of the LSVM, SVM with ANOVA, and LIBSVM with RBF is 86.11, 85.13, and 84.78%, respectively. The PPV for the SVM with ANOVA, LSVM, and LIBSVM with RBF models is obtained as 80.24, 77.21, and 76.24, respectively. In terms of the F-measure, sensitivity, specificity, and NPV criteria, the LSVM method has a better performance compared to the other two methods.

**Figure 10:**
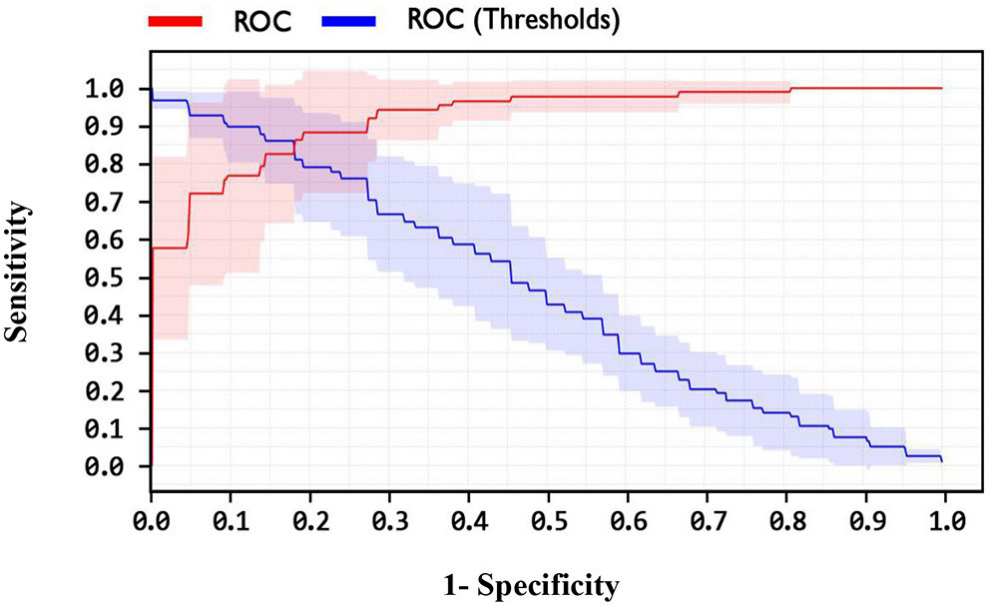
ROC curve for LSVM method.

**Figure 11:**
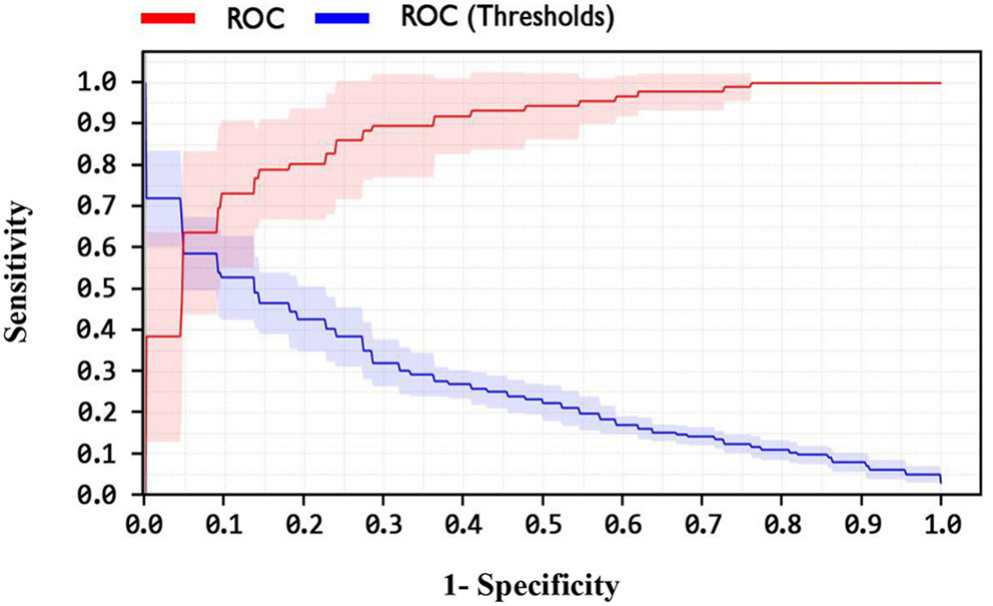
ROC curve for SVM with ANOVA method.

**Figure 12:**
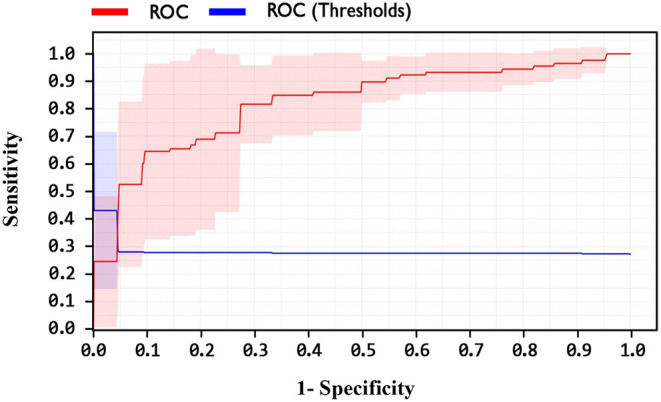
ROC curve for LIBSVM with RBF method.

In addition, one of the important criteria of the evaluation of methods is the AUC criterion. The AUC of methods was achieved as 92.4, 89, and 82.1% for LSVM, SVM with ANOVA, and LIBSVM with RBF methods, respectively. The ROC curve for the LSVM, SVM with ANOVA, and LIBSVM with RBF methods is shown in Figures 10–12, respectively.

By observing the Figures 10–12, it can be concluded that the LSVM method has better performance with an AUC of 92.4% than SVM with ANOVA and LIBSVM with RBF methods.

**Figure 13:**
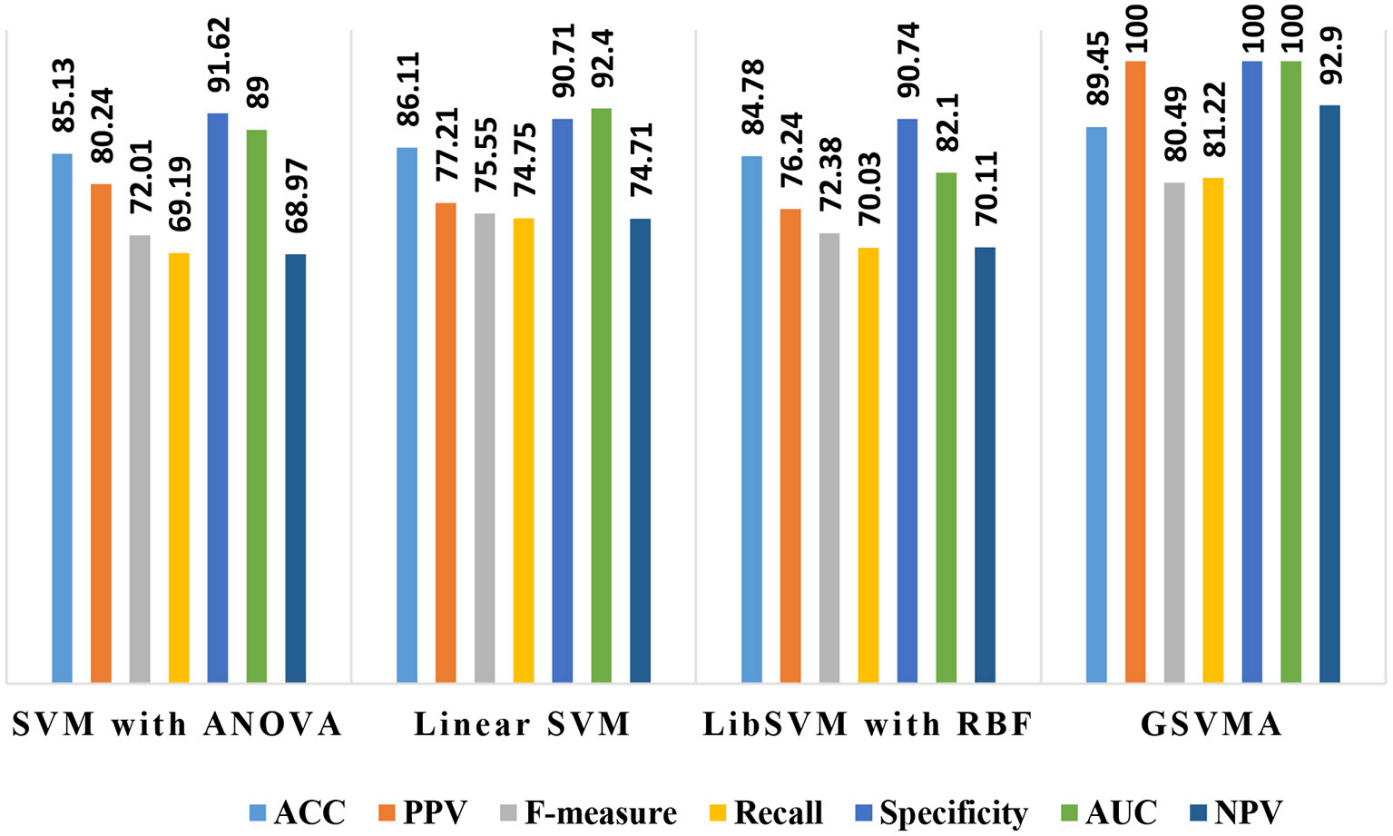
A comparison between the performance of methods based on the seven criteria.

Overall, the proposed GSVMA method has the best performance compared to the other methods in terms of accuracy, F-measure, PPV, NPV, sensitivity, specificity, and AUC. Figure 13 shows the comparison between the methods based on the seven criteria.

Moreover, according to the proposed method, out of 35 features, 31 features were selected using the genetic optimization algorithm. The crucial features include sex, CRF, CVA, airway disease, thyroid disease, CHF, systolic murmur, diastolic murmur, low TH ang, LVH, poor R progression, VHD, age, HTN, ex-smoker, FH, PR, typical chest pain, function class, Q wave, St elevation, T inversion, FBS, TG, LDL, ESR, lymph, Neut, PLT, EF-TTE, and region RWMA.

## Discussion

In this paper, we demonstrate the accuracy of 89.45% using the proposed GSVMA method for CAD diagnosis on the Z-Alizadeh Sani dataset by identifying 31 features. Finally, we compared our proposed method with the work of other researchers based on the Z-Alizadeh Sani dataset, as demonstrated in Table 8.

**Table 8:** Comparison between the proposed GSVMA method and the work of other researchers based on the original Z-Alizadeh Sani dataset.

**References**	**Techniques**	**No. of crossvalidation**	**No. of features**	**ACC (%)**	**AUC (%)**
Qin et al. (8)	Ensemble algorithm-multiple feature selection, (EA-MFS)	10-FCV	34	93.70 + −0.48%	NC
Cüvitoglu et al. (10)	Artificial neural network	10-FCV	25	87.85	93
Kiliç et al. (12)	Artificial Bee colony	-	16	89.4	NC
Abdar et al. (14)	NE-nu-SVC	10-FCV	16	94.66	NC
Abdar et al. (16)	N2Genetic-nuSVM	10-FCV	29	93.08	NC
Kolukisa et al. (17)	Ensemble classifier with Fisher linear discriminant analysis	5, 10, 20-FCV	55	92.07	95.3
Tama et al. (19)	Two-tier ensemble particle swarm optimization(PSO)-based feature selection	10-FCV	27	98.13	98.7
Terrada et al. (20)	(ANN)	-	17	94	94
Shahid et al. (22)	Hybrid PSO-EmNN	10-FCV	22	88.34	NC
Ghiasi et al. (23)	CART	10-FCV	5	92.41	NC
Dahal et al. (24)	SVM	10-FCV	15	89.47	88.68
Velusamy et al. (25)	Weighted-average Voting ensemble (WAVEn)	10-FCV	5	98.97	NC
Hassannataj et al. (5)	Random trees	10-FCV	40	91.47	96.7
The Proposed Method	GSVMA	10-FCV	31	89.45	100

*NC, Not considered*.

Based on Table 8, in the Qin et al. study (8), several feature selection methods had been implemented on the Z-Alizadeh Sani CHD dataset. The various assessment criteria to evaluate features coupled with a heuristic search strategy and seven classification methods are utilized. They further proposed an ensemble algorithm based on multiple feature selection (EA-MFS). The proposed EA-MFS method had better results with a reported accuracy of 93.70 and 95.53% F1-measure. In Cüvitoglu and Işik's study (10), an ensemble learner based on the combination of naïve Bayes, random forest, SVM, artificial neural networks (ANNs), and k-nearest neighbor algorithm is developed to diagnose CAD. Also, each of these methods is applied to the dataset separately. The authors performed a *t*-test for feature selection and reduced the feature space from 54 to 25. Moreover, they implemented PCA to reduce dimensionality further. Between the six methods, the best performance of the ANN achieved an AUC of 93%. Kiliç and Keleş (12) attempted to select the most convenient features to achieve better performance. They have used the artificial bee colony method on the Z-Alizadeh Sani dataset. The results showed that 16 of 56 features are more meaningful to predict CAD. They reported that a higher accuracy was achieved employing the selected features. Abdar et al. (14) proposed a model combining several traditional ML methods using ensemble learning techniques titled nested ensemble nu-support vector classification (NE-nu-SVC) to predict CAD. Also, they employed a feature selection routine based on a genetic algorithm and a filtering method to adjust data. The reported that accuracy of the NE-nu-SVC method is 94.66% for Z-Alizadeh Sani and 98.60% for Cleveland CAD datasets. In (16), Abdar et al. introduced the N2Genetic optimizer, which is a genetic-based algorithm and particle swarm optimization. Using the N2Genetic-nuSVM proposed, they achieved an accuracy of 93.08% and F1-score of 91.51% in the Z-Alizadeh Sani dataset for predicting CAD. In another study, Kolukisa et al. (17) examined two feature selection approaches to extract the most convenient set of features for the Z-Alizadeh Sani dataset. First, the features were selected based on medical doctor recommendations. According to clinically significant findings and Framingham heart study risk factors labeled features. The second method of feature selection was reported to improve the performance of ML algorithms. A combination of three ensemble learners, random forest, gradient boosting machine, and extreme gradient boosting, form a classifier to predict coronary heart disease in the work of Tama et al. (19). Moreover, a particle swarm optimization-based feature selection model takes the most valuable data features to feed the classifier efficiently. The functionality of the proposed system is verified by having Z-Alizadeh Sani, Statlog, Cleveland, and Hungarian datasets as the input data. The authors claim that the performance of their proposed model outperforms the present methods established on traditional classifier ensembles. They report a 98.13% accuracy, 96.60% F1-score, and 0.98 AUC to classify the Z-Alizadeh Sani dataset. The effectiveness of ANN and adaptive boosting algorithms to predict CAD was tested in the work of Terrada et al. (20). Data for this study were collected from Z-Alizadeh Sani, Hungarian, UCI repository, and Cleveland datasets, and 17 features were manually selected based on the atherosclerosis risk factors. The results indicated that ANNs show more promising performance over the adaptive boosting method. In (22), a hybrid algorithm based on emotional neural networks (EmNNs) and particle swarm optimization (PSO) is proposed by Shahid and Singh for CAD diagnosis. In addition, they implemented four unique feature selection techniques on the Z-Alizadeh Sani dataset to boost the functionality of the proposed model. Generally, their method has a better performance than the PSO-ANFIS model. The F1-score, accuracy, sensitivity, specificity, and PPV of the model are 92.12, 88.34, 91.85, 78.98, and 92.37%, respectively. According to Ghiasi et al. (23), only 40 independent parameters of the Z-Alizadeh Sani dataset affect the diagnosis of CAD. The authors apply the classification and regression tree (CART) method for this purpose. They further developed three additional CARD models utilizing 5, 10, and 18 selected features. For the developed model with five features, the reported accuracy is 92.41%. Also, a 77.01% true negative rate and 98.61% true positive value are reported for the model.

Dahal et al. (24) performed logistic regression, random forest, SVM, and K-nearest neighbors algorithms for CAD detection on the Z-Alizadeh Sani dataset to determine the most efficient technique. The results indicate that SVM has a better performance over other tested methods with 89.47% of accuracy. In (25), a study of CAD diagnosis was conducted using the weighted-average voting ensemble (WAVEn) method. Using this method, an accuracy of 98.97% was obtained on five features. Hassannataj et al. (5) used the random trees (RTs) on the 303 samples with 55 features. They have compared the RTs model with SVM, the C5.0 decision tree, and the CHAID decision tree. As a result, using the RTs model, 40 features were ranked with an accuracy of 91.47%, which RTs model has the best performance compared to the other models.

The results demonstrate the robustness of our proposed method in the diagnosis and prediction of CAD. Applying the GSVMA method, an accuracy of 89.45% was obtained by identifying 31 features. To the best of our knowledge, using this method, the AUC was achieved 100% on the original Z-Alizadeh Sani for the first time. Also, no previous works in the literature have investigated the NPV in CAD diagnosis, so that this criterion is essential in clinical practice. Despite these advances in the diagnosis of heart disease, there are limitations to the diagnosis process that we list below.

- Need a larger dataset to apply to the proposed GSVMA method.
- Lack of access to the real laboratory environment to record people's data in electronic profiles.
- Requiring the interaction of physicians and researchers to evaluate the results obtained properly.

## Conclusions

In this study, a hybrid method, namely GSVMA, is proposed to help the effective diagnosis and prediction of CAD by selecting essential features. This method was evaluated on the Z-Alizadeh Sani dataset. The GSVMA method consists of two main blocks. The first is the genetic optimization algorithm, in which essential features are selected by this algorithm. The second is the SVM algorithm with ANOVA kernel, which is used for classifying the input dataset. We carried out data preprocessing by converting nominal data to numerical data and performing a range transformation technique. Also, the 10-fold crossvalidation technique is used to split the dataset into two groups: 90% for training and 10% for testing. Moreover, other methods such as SVM with ANOVA, LSVM, and LIBSVM with RBF have been utilized to diagnose CAD. The proposed GSVMA method had the best performance compared to the mentioned methods regarding the accuracy of 89.45%, a PPV of 100%, a F-measure of 80.49%, a specificity of 100%, a sensitivity of 81.22%, a NPV of 92.9%, and an AUC of 100%, on 31 features among 55 features. By comparing the proposed method with related works, we found that the GSVMA method has good accuracy and AUC rates with the essential features. Besides, no previous works have studied the NPV in CAD diagnosis. In future work, if a larger dataset is available, the GSVMA method could be utilized. In addition, metaheuristic methods that include tabu search, iterated local search, simulated annealing, and variable neighborhood search can be used for feature selection. Then, each of these methods can be combined with machine learning methods.

## Data Availability Statement

The datasets presented in this study can be found in online repositories. The names of the repository/repositories and accession number(s) can be found at: https://archive.ics.uci.edu/ml/datasets/Z-Alizadeh+Sani, 20403598.

## Author Contributions

JH, FA, and RA designed the study. JH, FA, MN, and RA wrote the paper. JH, FA, MN, RA, and AM edited the paper. JH carried out the software on the Z-Alizadeh Sani dataset. JH, FA, RA, and IN generated all figures and tables. All authors have read and approved the final version of the paper.

## Funding

This work was supported by Alexander von Humboldt Foundation under project AvH0019272.

## Conflict of Interest

The authors declare that the research was conducted in the absence of any commercial or financial relationships that could be construed as a potential conflict of interest.

## Publisher's Note

All claims expressed in this article are solely those of the authors and do not necessarily represent those of their affiliated organizations, or those of the publisher, the editors and the reviewers. Any product that may be evaluated in this article, or claim that may be made by its manufacturer, is not guaranteed or endorsed by the publisher.

## Footnotes

^1^ https://archive.ics.uci.edu/ml/datasets/Z-Alizadeh+Sani
